# Long-term weight loss and metabolic benefit from Roux-en-Y gastric bypass in patients with superobesity

**DOI:** 10.1093/bjsopen/zrac145

**Published:** 2022-12-01

**Authors:** Styliani Mantziari, Theodoros Thomopoulos, Francesco Abboretti, Sergio Gaspar-Figueiredo, Anna Dayer, Nicolas Demartines, Michel Suter

**Affiliations:** Department of Visceral Surgery, Lausanne University Hospital (CHUV), Lausanne, Switzerland; Faculty of Biology and Medicine, University of Lausanne (UNIL), Lausanne, Switzerland; Department of Visceral Surgery, Lausanne University Hospital (CHUV), Lausanne, Switzerland; Department of Visceral Surgery, Lausanne University Hospital (CHUV), Lausanne, Switzerland; Department of Visceral Surgery, Lausanne University Hospital (CHUV), Lausanne, Switzerland; Department of Surgery, Hospital of Riviera Chablais, Rennaz, Switzerland; Department of Visceral Surgery, Lausanne University Hospital (CHUV), Lausanne, Switzerland; Faculty of Biology and Medicine, University of Lausanne (UNIL), Lausanne, Switzerland; Department of Visceral Surgery, Lausanne University Hospital (CHUV), Lausanne, Switzerland; Faculty of Biology and Medicine, University of Lausanne (UNIL), Lausanne, Switzerland; Department of Surgery, Hospital of Riviera Chablais, Rennaz, Switzerland

## Abstract

**Background:**

Although Roux-en-Y gastric bypass (RYGB) is widely performed worldwide, its efficacy in patients with a body mass index (BMI) greater than 50 kg/m^2^ remains controversial. The aim of the present paper was to assess long-term (10 years or more) weight loss and metabolic results of RYGB in patients with superobesity (SO; BMI > 50 kg/m^2^), compared with patients with morbid obesity (MO; BMI 35–50 kg/m^2^).

**Methods:**

This study involved retrospective analysis of a prospectively followed cohort of adult patients operated on for a primary RYGB between 1999 and 2008. Long-term weight loss and metabolic parameters were compared between SO and MO patients, with a sex-specific subgroup analysis in SO patients. Multiple logistic regression assessed independent predictors of poor long-term weight loss.

**Results:**

Among the 957 included patients, 193 (20.2 per cent) were SO (mean BMI 55.3 kg/m^2^*versus* 43.3 kg/m^2^ in MO). Upon 10-year follow-up, which was complete in 86.3 per cent of patients, BMI remained higher in SO patients (mean 39.1 kg/m^2^*versus* 30.8 kg/m^2^, *P* < 0.001) although total bodyweight loss (per cent TBWL) was similar (28.3 per cent *versus* 28.8 per cent, *P* = 0.644). Male SO patients had a trend to higher 10-year per cent TBWL, while initial BMI greater than 50 kg/m^2^ and low 5-year per cent TBWL were independent predictors of long-term TBWL less than 20 per cent. Diabetes remission was observed in 39 per cent SO and 40.9 per cent MO patients (*P* = 0.335) at 10 years, and all patients had a significant lipid profile improvement.

**Conclusion:**

Substantial improvement in co-morbidities was observed in all patients 10 years after RYGB. Total weight loss was similar in SO and MO patients, leaving SO patients with higher BMI. Suboptimal TBWL 5 years after surgery in SO, especially female patients, may warrant prompt reassessment to improve long-term outcomes.

## Teaser

In this series of 957 RYGB patients, 20.2 per cent had super-obesity (SO; BMI greater than 50 kg/m^2^). Substantial co-morbidity improvement was observed in all patients 10 years after RYGB. Total weight loss was similar in SO and MO patients, leaving SO patients with higher BMI; male patients had a trend to better long-term weight results.

## Introduction

The proportion of patients suffering from obesity is constantly rising worldwide^1^. According to the latest national health report in Switzerland, 42 per cent of the adult population is either overweight or obese^2^. In the meantime, the incidence of severe obesity is increasing; median BMI among patients undergoing bariatric surgery worldwide is estimated at 41.7 kg/m^2^, whereas it reaches 49.1 kg/m^2^ in Germany^3^.

Presently, bariatric surgery remains the standard for the treatment of morbid obesity (MO), as it provides superior weight and metabolic results and improves long-term life expectancy when compared with conservative methods^4,5^. Roux-en-Y gastric bypass (RYGB) is one of the most commonly performed bariatric procedures, with a proven lasting effect on weight control and metabolic profile improvement in patients suffering from obesity^6–12^. Nutritional deficiencies after RYGB are common and need rigorous follow-up and supplementation^13^, still they are rarely severe or refractory to treatment as opposed to those following malabsorptive procedures such as distal Roux-en-Y bypass (dRYGB)^14,15^, bilio-pancreatic diversion with/without duodenal switch (BPD-DS)^16^, or one-anastomosis gastric bypass (OAGB)^17^. This favourable risk–benefit balance makes RYGB the procedure of choice in many expert bariatric centres and explains probably why malabsorptive procedures represent only 1–2 per cent of all bariatric interventions performed annually^3^. Nevertheless, the efficacy of RYGB remains a matter of debate in patients with superobesity (SO) (BMI > 50 kg/m^2^). Some series present similar outcomes for patients with MO (BMI 35–50 kg/m^2^) and SO^18,19^, whereas others show inferior weight loss for SO patients^9,12,20–23^. Two studies suggested inferior weight loss in SO compared with MO patients more than 10 years after RYGB, although 10-year follow-up rates are rather poor (11.7–40.8 per cent)^9,12^.

As some well established (BPD-DS and dRYGB) and other more recent (OAGB and single anastomosis duodeno-ileal bypass (SADI)) malabsorptive procedures are often proposed to maximize weight loss, robust data are needed for the long-term effects of the standard RYGB procedure in SO patients. This is of particular clinical relevance as a two-step approach (sleeve gastrectomy followed by BPD-DS/OAGB/SADI-S, or even RYGB) is a valid option for SO patients, whereas upfront RYGB offers limited conversion strategies in cases where poor results are observed. There is, of course, the possibility of modifying limb length in RYGB (by elongating biliopancreatic (BP) limb of the Roux-en-Y bypass for example) to increase its efficiency; however, results are scarce concerning both weight loss benefits and potential metabolic complications, such as protein malnutrition^24^.

The aim of this study was to assess long-term weight loss and metabolic outcomes after RYGB in patients with SO compared with MO, and identify potential risk factors associated with suboptimal weight loss in the long term.

## Methods

All consecutive patients undergoing a primary laparoscopic RYGB between 1998 and 2008 in the two reference centres were included in a prospectively maintained database. They were divided into two groups according to their BMI at baseline: patients with SO and MO. The local ethics committee approved the study (protocol number 304/15), and consent was obtained from all patients for the use of clinical data for research purposes. The study was reported according to the STROBE guidelines for cohort studies^25^.

Surgical technique was standardized at both institutions during the study interval^8^, with a gastric pouch of 15 ml, anastomosed with a 21-mm circular stapler to a 150-cm retrocolic and retrogastric Roux-en-Y alimentary limb in SO patients and 100 cm in MO patients, except in 13 patients who had an antecolic Roux-en-Y limb. The jejuno-jejunostomy was performed with a side-to-side anastomosis at 30–50 cm from the angle of Treitz. Mesenteric windows (mesocolic, Petersen, and jejuno-jejunal) were closed (except in two early patients) using intermittent absorbable sutures for the first 209 patients, intermittent non-absorbable sutures for the next 171 patients, and running non-absorbable sutures for the remaining patients. Postoperative morbidity was recorded up to 30 days after surgery, and according to the Clavien–Dindo five-scale system^26^.

Weight loss results were assessed by means of absolute BMI, per cent total bodyweight loss from baseline (TBWL), and percentage of excess BMI loss (EBMIL), BMI = 25 kg/m^2^ being considered as the reference value. Although there is no universal agreement on what is considered ‘suboptimal weight loss’^27^, in the present study it was defined as less than 20 per cent TBWL 10 years after surgery^28^. Subgroup analyses were performed by sex, to assess potential differences in long-term outcomes in SO men and women. In terms of metabolic follow-up, the absolute values of glucose, triglycerides, total cholesterol, high-density lipoprotein (HDL) cholesterol, and low-density lipoprotein (LDL) cholesterol were prospectively recorded during the follow-up. Diabetes was diagnosed as fasting plasma glucose greater than 7 mmol/l, and impaired glucose tolerance as higher than 5.6 to less than 7.0 mmol/l, according to the American Diabetes Association guidelines^29^. As glycated haemoglobin (HbA1c) was not routinely measured during the study interval, diabetes remission was considered as complete normalization of fasting glucose levels without any medication, whereas diabetes improvement was defined as better control of diabetes with similar treatment, or similar control with reduced treatment^8^. Patient follow-up was conducted in the outpatient clinic where weight, co-morbidities, and blood test results were assessed. Patients who were eligible for 10-year follow-up but not seen for more than 12 months despite active tracking efforts, were considered lost from follow-up and were excluded from long-term weight and metabolic co-morbidity analysis.

Standard statistical comparisons were performed with the chi-squared or Fisher’s exact test for categorical variables, and the Mann–Whitney *U* test for continuous variables. Missing data were omitted from analyses, according to the default setting of the statistics software used. To determine factors independently associated with suboptimal weight loss, a multivariable logistic regression was performed. Co-variates with a *P* < 0.010 on a univariable level were included in the multivariable model, where *P* < 0.050 was the threshold for significance. Furthermore, a subgroup analysis of SO patients was performed to investigate the potential impact of sex on long-term outcomes. All analyses were performed with the R studio (version 1.1. 383, Boston, MA, USA) and SPSS^®^ (version 23.0, Chicago, IL, USA) software.

## Results

During the study interval, 957 patients underwent primary laparoscopic RYGB in the two participating centres and 193 of them (20.2 per cent) had a baseline BMI more than 50 kg/m^2^ (SO group). Of note, BMI more than 60 kg/m^2^ was observed in 33 (3.5 per cent) patients in this series. A complete 10-year follow-up was available for 86.3 per cent of all patients.

Baseline characteristics of all patients are summarized in *Table 1*. Male sex was more prevalent in the SO group (32.6 per cent *versus* 22.4 per cent *P* = 0.011). In addition, male SO patients had a poorer metabolic profile (hypertension, coronary artery disease, and hypertriglyceridemia) (*Table 2*). Although operating time (160 *versus* 144 min, *P* < 0.001) and length of hospital stay (6.1 *versus* 4.6 days, *P* = 0.045) were significantly longer for SO patients, postoperative outcomes were similar (*Table 3*). During long-term follow-up, there were no differences in internal hernia incidence or any other surgical complications (*Table 4*).

**Table 1:** Baseline demographic characteristics and co-morbidities for all patients

All patients	SO *n* = 193	MO *n* = 764	*P*
Age (years), mean(s.d.)	40.2(10.8)	40.0(10.7)	0.810
Weight (kg), mean(s.d.)	153.9(23.1)	119.7(15.3)	<0.001
BMI (kg/m^2^), mean(s.d.)	55.3(5.3)	43.3(3.0)	<0.001
Sex ratio (M:F)	63 (32.6):130 (67.4)	171 (22.4):573 (77.6)	0.011
Diabetes	118 (61.1)	424 (55.5)	0.295
Hypertension	117 (60.6)	407 (53.3)	0.017
Coronary artery disease	10 (5.2)	27 (3.5)	0.256
Hypercholesterolaemia	113 (58.5)	513 (67.1)	0.021
Hypertriglyceridaemia	65 (33.7)	301 (39.4)	0.025
Hyperuricaemia	70 (36.3)	250 (32.7)	<0.001
Osteoarticular pain	129 (66.8)	547 (71.6)	0.223
Sleep apnoea syndrome	124 (64.2)	338 (44.2)	<0.001
Gastroesophageal reflux	87 (45.1)	400 (52.4)	0.005
Depression	39 (20.2)	163 (21.3)	0.676

Values are *n* (%) unless otherwise indicated Mean(s.d.) age of patients was 40 years, with a BMI of 55.3(5.3) kg/m^2^ in SO and 43.3(3) kg/m^2^ in the MO group. SO, superobesity; MO, morbid obesity; BMI, body mass index.

**Table 2:** Baseline demographic characteristics and co-morbidities for male and female patients with superobesity

SO subgroup	Male SO *n* = 63	Female SO *n* = 130	*P*
Age (years), mean(s.d.)	40.4(10.6)	40.1(10.9)	0.854
Weight (kg), mean(s.d.)	172.8(22.2)	144.8(17.2)	<0.001
BMI (kg/m^2^), mean(s.d.)	56.1(6.2)	55.0(4.8)	0.185
Diabetes	40 (63.5)	78 (60)	0.131
Hypertension	45 (71.4)	72 (55.4)	<0.001
Coronary artery disease	7 (11.1)	3 (2.3)	0.009
Hypercholesterolaemia	35 (55.6)	78 (60.0)	0.070
Hypertriglyceridaemia	25 (39.7)	40 (30.8)	0.007
Hyperuricaemia	27 (42.9)	43 (33.1)	0.133
Osteoarticular pain	38 (60.3)	91 (70.0)	0.407
Sleep apnoea syndrome	50 (79.4)	74 (56.9)	<0.001
Gastroesophageal reflux	35 (55.6)	52 (40.0)	0.092
Depression	8 (12.7)	31 (23.8)	0.195

Values are *n* (%) unless otherwise indicated. SO, superobesity; MO, morbid obesity; BMI, body mass index.

**Table 3:** Postoperative outcomes in patients with superobesity and morbid obesity

	SO *n* = 193	MO *n* = 764	*P*
**Anastomotic leak**	5 (2.6)	10 (1.3)	0.200
Gastrojejunostomy	2	8	1.000
Jejuno-jejunostomy	1	1	0.362
Gastric remnant	2	3	0.262
**Surgical site infection**	8 (4.1)	34 (4.4)	1.000
Superficial	6	23	0.999
Deep	3	10	0.732
**Haemorrhagic complications**	7 (3.6)	27 (3.5)	0.954
**Venous thromboembolic events**	3 (1.6)	10 (1.3)	0.733
**Overall morbidity rate**	21 (10.9)	90 (11.8)	0.801
**Major complications (more than Clavien score IIIA)**	6 (3.1)	22 (2.9)	0.813
**Operative duration (min), mean(s.d.)**	160(42.2)	143(38.2)	<0.001
**Postoperative duration of hospital stay (days), mean(s.d.)**	6.1(7.2)	4.7(3.9)	0.005

Values are *n* (%) unless otherwise indicated. SO, superobesity; MO, morbid obesity.

**Table 4:** Long-term complications and reoperations in patients with superobesity and morbid obesity

	SO *n* = 193	MO *n* = 764	*P*
Intestinal obstruction	7 (3.6)	38 (4.9)	0.562
Internal hernia	12 (6.2)	60 (7.8)	0.540
Marginal ulcer	2 (1)	12 (1.5)	0.743
Incisional hernia	2 (1)	5 (0.6)	0.629
Recurrent abdominal pain	5 (2.6)	20 (2.6)	1.013
Anastomotic stricture	6 (3.1)	33 (4.3)	0.540
Intussusception	0	3 (0.4)	1.042
Hiatus hernia	0	4 (0.5)	0.581
Candy cane	1 (0.5)	5 (0.6)	1.005
Symptomatic gallstones	2 (1)	2 (0.3)	0.184
Patients requiring reoperation	21 (10.8)	105 (13.7)	0.341
Patients requiring endoscopic dilatation	7 (3.6)	34 (4.4)	0.688

Values are *n* (%) unless otherwise indicated. SO, superobesity; MO, morbid obesity.

### Long-term weight loss results in superobesity and morbid obesity patients

**Fig. 1:**
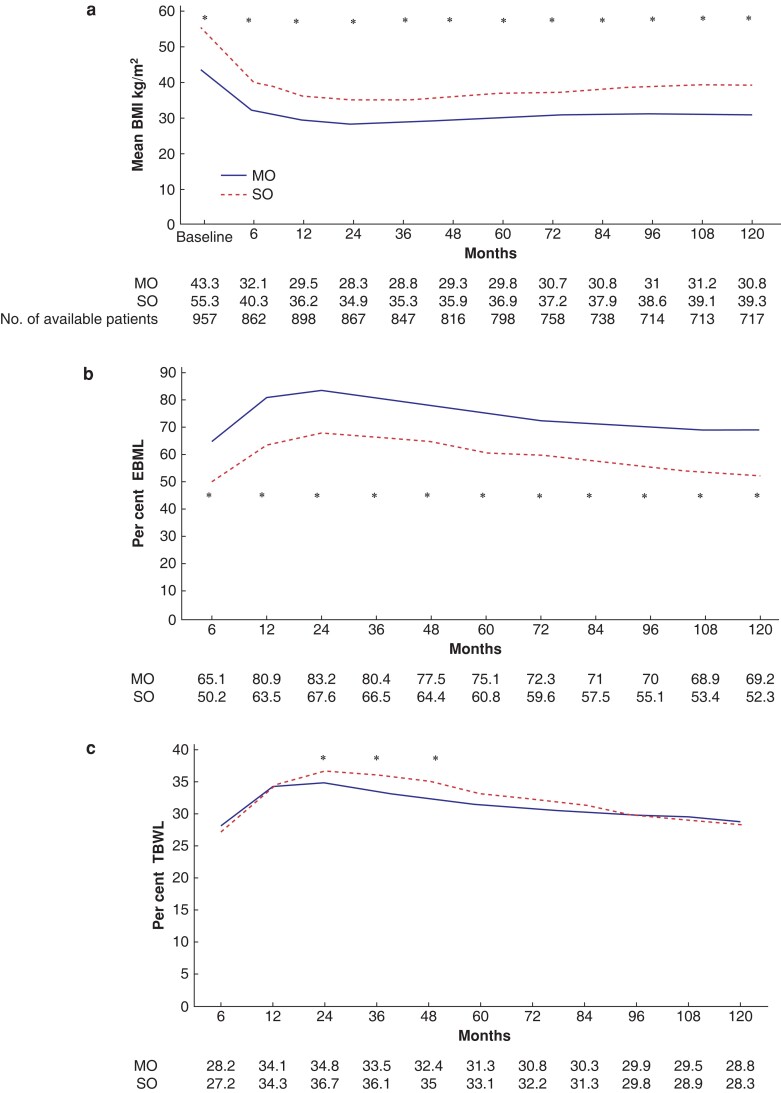
Weight evolution of patients with morbid obesity *versus* superobesity during the 10-year follow-up **a** BMI values. **b** Excess BMI loss (EBMIL per cent). **c** Total bodyweight loss (TBWL per cent). All variables are represented by mean values in each time point. An asterisk (*) indicates significant a difference between MO and SO patients. SO, superobesity; MO, morbid obesity; BMI, body mass index.

All patients lost similar proportions of their initial weight at 10-year follow-up (TBWL 28.3 per cent for SO and 28.8 per cent for MO patients, *P* = 0.644). Between 24 and 48 postoperative months SO patients had a significantly higher %TBWL, although no difference was observed from the fifth year on. At 10 years, mean BMI was 39.3 kg/m^2^ and 30.8 kg/m^2^ respectively (*Fig. 1a–c*). ‘Suboptimal weight loss’ (TBWL less than 20 per cent) was observed in 37 (25.3 per cent) of SO and 107 (17.8 per cent) of MO patients 10 years after surgery (*P* = 0.037). At 10 postoperative years, 84 (57.5 per cent) patients in the SO group and 580 (96.8 per cent) in the MO group had a BMI less than 40 kg/m^2^; 51 (34.9 per cent) SO and 490 (82.1 per cent) MO patients had a BMI less than 35 kg/m^2^, whereas 9.6 per cent SO and 44.4 per cent MO patients achieved a BMI less than 30 kg/m^2^ (*P* < 0.001 for all comparisons).

Multivariable analysis revealed BMI greater than 50 kg/m^2^ (SO group) at baseline (OR 1.94, 95 per cent c.i. 1.01 to 3.70, *P* = 0.044) and low %TBWL at 5 postoperative years (OR 0.80, 95 per cent c.i. 0.76 to 0.85, *P* < 0.001) as the only independent predictors of suboptimal 10-year weight loss (*Table S1*).

### Metabolic results in superobesity and morbid obesity patients

**Fig. 2:**
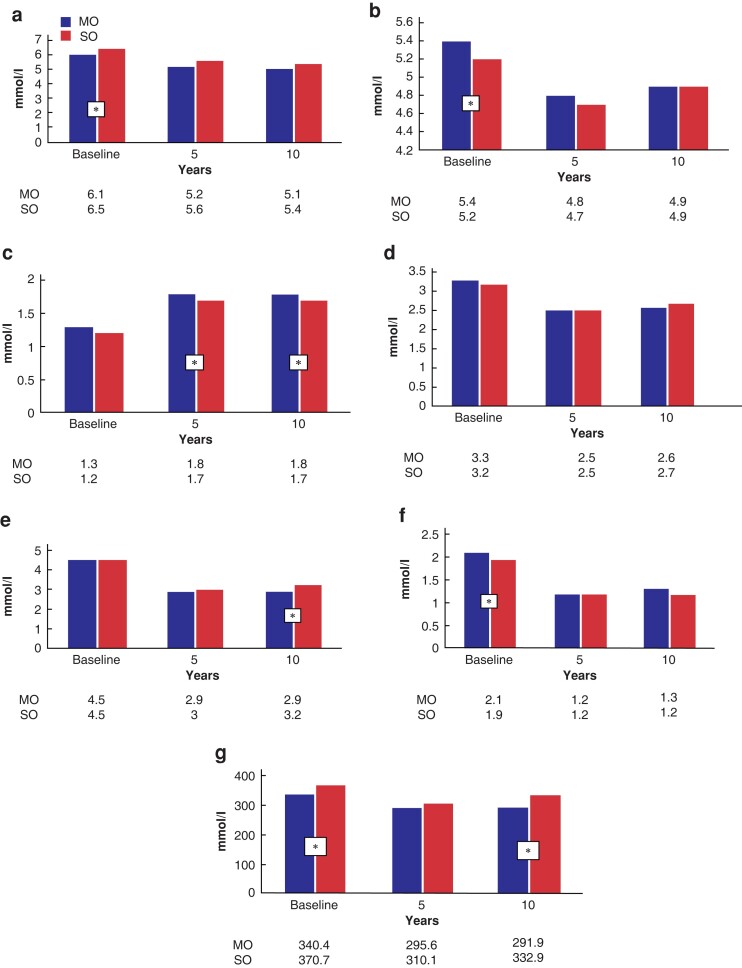
Metabolic profile evolution of patients with morbid obesity *versus* superobesity during the 10-year follow-up **a** Fasting glycaemia values (mol/l). **b** Total cholesterol (mmol/l). **c** High-density lipoprotein (HDL) cholesterol (mmol/l). **d** Ratio total/HDL cholesterol. **e** Low-density lipoprotein (LDL) cholesterol (mmol/l). **f** Triglycerides (mmol/l). **g** Urates (mmol/l). All variables are represented by mean values. An asterisk (*) indicates significant a difference between MO and SO patients. SO, superobesity; MO, morbid obesity.

Ten years after RYGB, 57 (39.0 per cent) SO patients and 244 (40.9 per cent) MO patients initially suffering from diabetes mellitus (DM), presented complete diabetes remission (*P* = 0.335). Inversely, 4 (2.7 per cent) SO and 32 (5.4 per cent) MO patients presented *de novo* diabetes (*P* = 0.335). Mean fasting glucose levels were higher at baseline for the SO group; however, the difference disappeared at 10 postoperative years (*Fig. 2a*). Patients with suboptimal weight loss (less than 20 per cent TBWL) at 10 years had inferior rates of complete diabetes remission (36.7 per cent *versus* 40.1 per cent, *P* = 0.029). Evolution of lipid profile is shown in *Fig. 2b–f*, with MO patients presenting higher HDL and lower total cholesterol/HDL ratio 10 years after surgery. Uric acid levels remained higher for SO patients throughout the 10-year follow-up (*Fig. 2g*).

Of note, 10-year all-cause mortality rate was 5.7 per cent in SO and 2.1 per cent in the MO patients (*P* = 0.012).

### Sex-specific weight results in superobesity patients

**Fig. 3:**
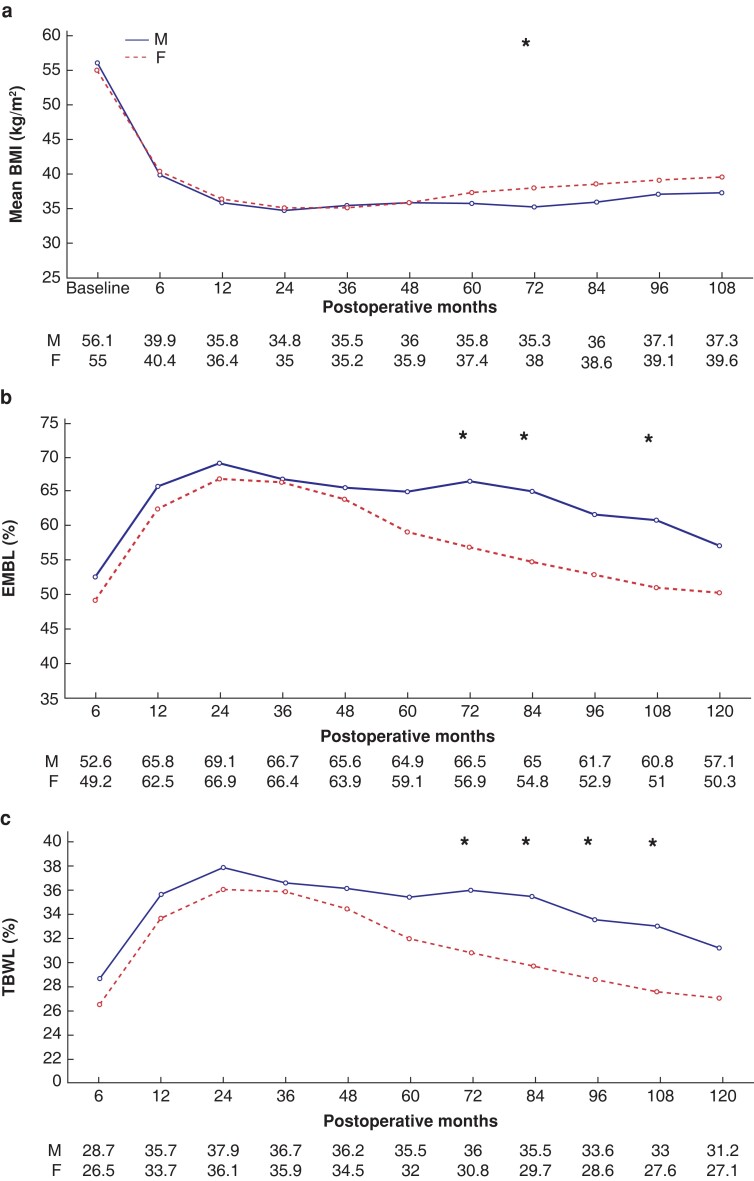
Weight evolution of male *versus* female patients with superobesity during the 10-year follow-up **a** BMI values. **b** Excess BMI loss (EBMIL per cent). **c** Total bodyweight loss (TBWL per cent). All variables are represented by mean values in each time point. An asterisk (*) indicates significant a difference between male and female patients. M, male; F, female; BMI, body mass index.

Baseline characteristics of SO male and female patients are shown in *Table 2*. No difference in operating time, postoperative complications, or length of stay were observed between male and female SO patients (data not shown). Mean BMI remained similar up to the fifth postoperative year, when females started regaining more weight (*Fig. 3a–b*). Male SO patients showed a trend to higher per cent TBWL from the fifth and up to the 10th postoperative year, with significantly better results between 72 and 108 postoperative months (*Fig. 3c*). At 10 years, five (12.2 per cent) male and 32 (30.5 per cent) female SO patients presented poor weight loss (*P* = 0.022), whereas five (12.2 per cent) male and nine (8.6 per cent) female patients achieved a BMI less than 30 kg/m^2^ (*P* = 0.504).

## Discussion

In the present series of RYGB, patients with SO represented 20 per cent of all cases. Although they had similar TBWL as patients with MO 10 years after surgery, preoperative BMI more than 50 kg/m^2^ was independently associated with suboptimal long-term weight loss. Female SO patients presented lower weight loss compared with male SO patients at 10 postoperative years. All patients had similar rates of DM remission at 10 years and managed to improve their lipid profile.

Preliminary mid-term institutional data (five postoperative years) suggested that although SO patients achieve similar or even higher absolute weight loss (BMI units, kg) than MO patients, their BMI tends to remain higher^21^. Therefore, when results are expressed with metrics referring to an ideal weight (per cent Excess Weight Loss (EWL), per cent EBMIL, and BMI), they are largely dependent on baseline BMI. In the present study^28,30^, a 10-year TBWL less than 20 per cent was chosen to define suboptimal weight loss, as TBWL is the least influenced from baseline BMI and of great clinical relevancy, as patients’ perception of weight loss ‘success’ is largely based on their own preoperative status, and not on ideal weight references.

Although there was no significant difference in mean 10-year %TBWL between SO and MO groups, a higher proportion of SO patients achieving suboptimal weight loss in the long term was found. Previously, Christou *et al.*^9^ reported 10-year rates of suboptimal weight loss in 34.9 per cent (SO) and 20.4 per cent (MO) patients using the Biron criteria (BMI more than 35 kg/m^2^ for MO and BMI more than 40 kg/m^2^ for SO patients)^31^, whereas Magro *et al.* reported 20 per cent and 10.1 per cent in SO and MO patients, when more than 50 per cent EWL was used as a cut-off^20^; however, some further insight is needed in interpreting when defining ‘successful’ weight loss after bariatric surgery, as there is no universally accepted weight loss cut-off predicting co-morbidity evolution and patient satisfaction^14,20^. Obeid *et al.* illustrated that despite the difference in per cent EWL between SO (52.9 per cent) and MO patients (61.3 per cent), obesity-related co-morbidities were significantly improved in all patients a decade after RYGB^12^. In the present study, SO and MO patients had comparable rates of DM remission at 10 years, approximating 40 per cent of all patients who had DM initially, whereas a low rate of *de novo* DM was noted in both groups. This, along with the sustained improvement in lipid profile observed in all patients, confirms that a weight loss-independent metabolic benefit is seen after RYGB^6,11^ contributing to the subsequent reduction in cardiovascular mortality^32,33^. Of note, long-term surgical complications were comparable between SO and MO patients in the present series, but a significantly higher long-term mortality was confirmed in the SO population; this illustrates the deleterious impact of severe obesity on long-term survival.

One might argue that to deal with the massive weight excess in SO patients, more malabsorptive procedures than the standard (proximal) RYGB should be preferred. Although Brolin *et al.* had suggested better results for more distal RYGB^34^, Risstad *et al.* did not find superior weight loss after distal *versus* proximal RYGB in these patients^14^. Co-morbidities and specifically diabetes were well controlled in both groups, whereas distal RYGB patients had a significantly worse quality of life and social limitations due to loose stool and malabsorption^14^. In another RCT comparing RYGB with BPD-DS, 55.6 per cent of SO patients had suboptimal weight results 5 years after RYGB, compared with 14.3 per cent after BPD-DS^16^. These results corroborate with older data suggesting superior weight loss after BPD-DS in SO patients^35^; however, overwhelming diarrhoea, severe hyperparathyroidism, protein malnutrition, and even liver failure were exclusively reported after BPD-DS^16^. In addition, although dRYGB^14^ and BPD-DS^16^ yielded better fasting glucose and HbA1c values than standard RYGB in SO patients, all markers remained well under the diabetes threshold for RYGB, dRYGB, and BPD-DS patients. The present study confirms a sustained TBWL of 28 per cent for SO patients 10 years after RYGB, which remains in the upper range of the reported 22.5–31.6 per cent for the general RYGB population in the literature^6,8,11,12,23^. Still, 25.3 per cent SO patients (and up to 30.5 per cent among women) presented suboptimal weight loss (TBWL less than 20 per cent) 10 years after surgery; these patients had also inferior rates of DM remission. Thus, a more aggressive bariatric approach is worth discussing in cases of extreme obesity. Bolckmans *et al.* reported 40.7 per cent TBWL 10 years after BPD-DS^36^; another series suggested similar 5-year weight loss after OAGB and RYGB (40.8 per cent *versus* 37.2 per cent respectively), with comparable rates of diabetes remission^37^. In a recent case-match study of patients with severe obesity, SADI-S presented superior mid-term (more than 5 years) surgical outcomes as well as weight control than RYGB patients^38^. A recent meta-analysis assessed current options in patients with weight regain after primary RYGB, suggesting dRYGB as the most efficient solution to tackle weight regain, followed by BPD/DS and SADI-S^39^; however, robust data on long-term metabolic complications and patient-reported outcomes are still lacking; weight loss expectations need to be put in a realistic and clinically relevant perspective when counselling SO patients, considering the potentially invalidating side effects of malabsorptive procedures for the sake of supplementary weight loss.

This is one of the first studies reporting more favourable weight loss outcomes after RYGB in male SO patients compared with females. Although a robust pathophysiological explanation cannot be provided based on our results, the loss of lean mass in association with low oral protein intake may contribute to lower resting energy expenditure in female patients after bariatric surgery^40^. As detailed data on body composition, dietary habits, and exercise are not available in the present series, this sex-specific analysis can only be considered hypothesis-generating.

Multivariate analysis confirmed an SO status and %TBWL at 5 years as independent predictors of suboptimal weight loss at 10 years. Previous long-term series reported maximal weight reduction during the first 2–5 years after RYGB, followed by a phase of weight maintenance or regain up to the 10th year^6,9,11,12,20^; however, up to 10 per cent of patients may achieve their minimal weight 10 years after surgery^8^. Even so, patients with suboptimal weight loss in the mid-term warrant close attention. Nutritional counselling, behavioural treatment, and a thorough assessment of the patient’s co-morbidities and functional status need to be undertaken to halt or reverse weight regain^15^.

This study has some limitations. Although weight, metabolic biomarkers, and co-morbidities were prospectively recorded for all patients, other relevant outcomes such as nutritional deficiencies and compliance to supplementation were not systematically documented in the early years of our prospectively followed cohort. In addition, patient-reported outcomes were not systematically collected, so the actual weight loss ‘failure’ cannot be correlated with patients’ perception. These shortcomings are counterbalanced by the large number of included patients, the homogeneity of surgical management over the years, as well as the 86.3 per cent complete 10-year follow-up of the cohort, which is one of the highest reported in the bariatric literature. Moreover, the standard laparoscopic Roux-en-Y technique performed in this non-selected cohort of consecutive primary RYGB cases, allows for a safe extrapolation of the current results into general practice.

In conclusion, TBWL in SO is comparable to that in MO patients 10 years after RYGB, leaving SO patients with higher BMI values. Suboptimal outcomes 5 years after surgery in SO, especially in female patients, could warrant a multidisciplinary intervention to evaluate if, and by which means, the course of obesity can still be changed.

## Supplementary Material

zrac145_Supplementary_Data

## Data Availability

The authors declare that their data, analytic methods, and study materials may be made available to other researchers, upon request to the corresponding author.
