# Supplementary material for: Long-term weight loss and metabolic benefit from Roux-en-Y gastric bypass in patients with superobesity
Source: BJS Open. 2022 Dec 1;6(6):zrac145. doi: 10.1093/bjsopen/zrac145 (PMC9713229; doi:10.1093/bjsopen/zrac145)
Supplement: zrac145_Supplementary_Data [file zrac145_supplementary_data.docx]

**Table S1.**  Logistic regression for suboptimal weight loss (TBWL<20%) at 10 postoperative years

|  | Unadjusted OR | 95%CI | *P-value* | Adjusted OR | 95%CI | *P-value* |
| --- | --- | --- | --- | --- | --- | --- |
| Age | 0.99 | 0.97-1.01 | 0.199 |  |  |  |
| Gender  Female  Male | 1.45 | 0.95-2.18 | 0.075 | 1  0.92 | 0.50-1.65 | 0.787 |
| SO at baseline | 1.57 | 1.01-2.39 | 0.039 | 1.94 | 1.01-3.70 | 0.044 |
| Hypertension | 1.04 | 0.72-1.50 | 0.839 |  |  |  |
| Diabetes | 0.80 | 0.55-1.16 | 0.244 |  |  |  |
| Coronary artery disease | 0.82 | 0.27-2.02 | 0.700 |  |  |  |
| Hypertriglyceridemia | 1.45 | 0.99-2.11 | 0.053 | 1.15 | 0.67-1.94 | 0.612 |
| Depression | 0.97 | 0.62-1.49 | 0.906 |  |  |  |
| Osteoarticular pain | 0.82 | 0.55-1.26 | 0.357 |  |  |  |
| TBWL (%)  6 months postop  24 months postop  60 months postop | 0.93  0.86  0.80 | 0.90-0.97  0.83-0.89  0.77-0.83 | 0.0005  <0.001  <0.001 | 1.02  1.01  0.80 | 0.98-1.05  0.96-1.07  0.76-0.85 | 0.308  0.609  <0.001 |

OR=Odds Ratio, 95%CI=95% Confidence Intervals, SO=Superobese, TBWL= Total Body Weight Loss (%)
